# Association between School Contexts and the Development of Subjective Well-Being during Adolescence: A Context-Sensitive Longitudinal Study of Life Satisfaction and School Satisfaction

**DOI:** 10.1007/s10964-022-01727-w

**Published:** 2023-01-19

**Authors:** Yi-Jhen Wu, Michael Becker

**Affiliations:** 1 Center for Research on Education and School Development, Technical University Dortmund, Dortmund, Germany; 2 DIPF|Leibniz Institute for Research and Information in Education, Frankfurt, Germany

**Keywords:** Life satisfaction, School satisfaction, Secondary schooling, Ability grouping, Achievement composition, Longitudinal study

## Abstract

The transition to secondary school may negatively impact adolescents’ psychosocial and subjective well-being development. However, how subjective well-being develops during secondary school and how school contextual factors, including aspects of ability grouping and achievement composition, are associated with the development of subjective well-being still require clarification. This study examined two measures of subjective well-being, life satisfaction and school satisfaction, to investigate the development of subjective well-being during secondary school. Moreover, school context variations in the form of school tracks and school-level achievement were analyzed to examine the extent to which ability grouping and achievement composition were associated with the development of subjective well-being. A large-scale longitudinal German dataset with four measurement points from grades 6 to 10 was analyzed (Time 1: *N* = 1,841; *M*_age_ = 12.20, *SD* = 0.81; 48.4% female; 45.3% immigrant students). The latent growth model revealed that life satisfaction and school satisfaction decreased statistically significantly during secondary school, yet school satisfaction showed a temporary increase between the end of primary school and right after the transition to secondary school. School tracks did not statistically significantly predict the magnitude of the decline in life satisfaction or school satisfaction. Only school-level achievement composition significantly negatively predicted the decline in life satisfaction, suggesting that students in schools with higher levels of achievement composition had a greater decrease in life satisfaction than their counterparts in schools with lower levels of achievement composition. Taken together, these findings contribute to the knowledge of how life and school satisfaction develop during secondary school and the long-term associations between subjective well-being and school context factors.

## Introduction

Understanding how adolescents’ subjective well-being develops during the transition to secondary school is paramount because the transition to secondary school poses many challenges for adolescents’ development (Loukas et al., 2016; Griffith et al., 2021; Wang & Eccles, 2012). The Stage-Environment-Fit model posits that misfit between developmental needs and a secondary school environment might hinder adolescent development (Eccles et al., 1993). Although a decline in life satisfaction during secondary school has been found (Casas & González-Carrasco, 2019; Daly, 2022), it is unclear whether that decline is associated with the transition to secondary school, as life satisfaction involves many life facets. To understand whether the transition to secondary school has a negative impact on adolescents’ subjective well-being, it seems necessary to consider not only life satisfaction in general, but also school satisfaction, particularly as it ties closer to school contexts. However, no study has jointly investigated life satisfaction and school satisfaction to provide broader evidence of the development of subjective well-being during the transition to secondary school. Furthermore, from a socio-ecological perspective, adolescent development is embedded in a complex school environment (Bronfenbrenner, 1977). The most prominent school contextual factors discussed in psychosocial development are ability grouping and achievement composition (Marsh et al., 2008), which are closely related. These contextual factors might shape different school experiences for adolescents, which might influence their judgment of subjective well-being (Baumert et al., 2010; Harris, 2010; Schofield, 2010). However, the long-term prediction of ability grouping and achievement composition on subjective well-being remain unclear. Thus, the current study aimed to investigate the development of life satisfaction and school satisfaction during secondary school and their longitudinal associations with school contextual factors, including ability grouping and achievement composition.

### Development of Global and Domain-Specific Subjective Well-Being during Secondary Schooling

Subjective well-being refers to individual cognitive evaluations toward life experiences, positive (e.g., joy, optimism) and negative affect (e.g., sadness, anger, anxiety) in one’s specific life domains, and general life (Diener, 1984). Subjective well-being typically uses life satisfaction as global subjective well-being to measure all aspects of life. Life satisfaction can provide evidence for the general development of adolescents; unfortunately, the development of life satisfaction may not necessarily be associated with school context. To fully understand how secondary school is associated with the development of subjective well-being, the most important domain-specific measure is school satisfaction. School satisfaction is defined as the judgment of overall school life (Huebner, 1994). By definition, it maps directly into many aspects of adolescents’ school lives, which might reflect changes in the school environment during the transition to secondary school.

In general, the transition to secondary school poses negative consequences on adolescents’ psychosocial and subjective well-being development. The Stage-Environment Fit model posits that a secondary school environment might not provide appropriate opportunities to fulfill adolescents’ basic psychological needs related to competence, relatedness, and autonomy, resulting in person-environment misfit (Eccles et al., 1993). Previous studies have found that person-environment misfit was associated with declining patterns in academic and general psychosocial variables (Engels et al., 2017; Scherrer & Preckel, 2019). If misfit contributes to a decline in a wide range of psychosocial variables, it might be expected that life and school satisfaction decline during secondary school. Inferences can be drawn from studies focusing on positive and negative affect, which are the most relevant constructs to life satisfaction. Research has found that positive affect decreased and negative affect increased during secondary school (Griffith et al., 2021; Grills-Taquechel et al., 2010; Meyer & Schlesier, 2022). Most studies have found that life satisfaction decreased during secondary school (Casas & González-Carrasco, 2019; Daly, 2022; Proctor et al., 2009). Although a few studies have demonstrated that life satisfaction increased during the middle to later periods of adolescence, the different findings might be associated with different stages of adolescence (Salmela-Aro & Tuominen-Soini, 2010; Steinmayr et al., 2019; Willroth et al., 2021).

Whether school satisfaction declines during secondary school is unclear. According to specificity-matching principles (Swann et al., 2007), a domain-specific construct should predict another domain-specific construct more strongly than a global construct. Following this reasoning, one might expect that school satisfaction is more likely to reflect misfit between the secondary school environment and adolescents’ needs, as school satisfaction is closer to many specific aspects of school life than general life satisfaction. Previous studies have demonstrated that academic-specific psychosocial variables, including self-beliefs and motivation, declined during secondary school (Scherrer & Preckel, 2019 for review). Furthermore, studies using a similar construct to school satisfaction found that school attitudes, connectedness, and emotional engagement decreased during secondary school (Booth & Gerard, 2014; Engels et al., 2017; Li & Lerner, 2011; Loukas et al., 2016; Wang & Eccles, 2012). As current evidence consistently demonstrates that academic-related psychosocial variables and similar constructs to school satisfaction decline during secondary school, this might also imply that school satisfaction declines.

However, previous empirical studies only provided evidence regarding the declining patterns of life satisfaction and school satisfaction rather than the *shape* of development. Life satisfaction is the aggregate of various facets of domain-specific satisfaction. This definition suggests a bottom-up perspective that individuals evaluate domain-specific satisfaction separately, resulting in an overall rating of life satisfaction (Diener, 1984; McAdams et al., 2012). This perspective implies that the developmental shape of life satisfaction might not necessarily be similar to domain-specific satisfaction. A large cross-sectional study in an adult population found that only some domain-specific satisfaction, including family and job satisfaction, exhibited developmental shapes similar to life satisfaction across the lifespan when domain-specific satisfaction had substantial predictions of life satisfaction (Easterlin, 2010). Another lifespan study further found that each domain-specific satisfaction showed quite different developmental shapes compared to life satisfaction (McAdams et al., 2012). Considering theoretical and empirical evidence, one might expect that the developmental shape of life satisfaction might differ from school satisfaction, although both might decline during secondary school.

Although previous studies have attempted to understand the development of subjective well-being during secondary school, most have been limited to life satisfaction. Not knowing the trajectories of school satisfaction might oversimplify the development of subjective well-being, as school satisfaction might be more connected to the school environment. Although previous studies have suggested declining patterns of life satisfaction and school satisfaction, the potential developmental shapes of life satisfaction and school satisfaction are unknown. Thus, there is a strong need to jointly investigate the trajectories of life and school satisfaction to provide more nuanced evidence of how the development of subjective well-being and how is related to the transition to secondary school.

### The Associations between Development of Subjective Well-Being and School Context: Ability Grouping and Achievement Composition

#### Ability grouping

Ability grouping is one of the most important secondary school features in influencing adolescent psychosocial development (Birkelund, 2021; Frenzel et al., 2007; Marsh et al., 2008). Ability grouping has been implemented in secondary schools worldwide, and uses students’ academic abilities to assign them to different learning environments (Baumert et al., 2010; Schofield, 2010). The theoretical rationale of ability grouping is to facilitate instruction and learning by creating more homogeneous groups of students, so that teachers can tailor instruction to optimize students’ learning outcomes. Ability grouping—oftentimes also labeled “ability tracking” or “tracking” in general^1^—can be classified into three general types: (1) between-school, (2) within-school, and (3) course-by-course tracking (Chmielewski et al., 2013). Between-school tracking sorts students into different schools, creating distinct learning environments across ability groups. Within-school tracking consistently assigns students across subjects to different tracks, programs, or streams within a school. Finally, the least systematic practice of ability grouping is course-by-course, which assigns students to different course levels for specific domains and subjects in a school.

In this study, between-school tracking was the main focus. First, as between-school tracking shapes relatively more systematic and structured differences in learning and social environments than other types of tracking, differences between learning environments should be stressed. As a result, the practice of between-school tracking provides a stronger test of how ability grouping is associated with the development of subjective well-being. The practice of ability grouping typically reflects variations in institutional structures—different curricula and learning goals, levels of teachers’ professional training, and student compositions—thereby creating distinct learning and social environments (Baumert et al., 2010; Harris, 2010; Schofield, 2010). Learning goals in academic tracks generally focus on higher academic qualifications. Academic tracks generally provide advanced knowledge in disciplines, more cognitively activated instruction, demanding learning tasks, and teachers with higher levels of professional training. On the other hand, nonacademic tracks are generally less academic-oriented environments that provide a broad level of knowledge in disciplines, less cognitively demanding instruction and workloads, and teachers with less subject-specific professional training (Baumert et al., 2010). These different characteristics of learning environments might determine the extent to which school tracks would provide opportunities and constraints to students, which is inextricably linked to psychosocial and subjective well-being development.

Second, previous studies related to subjective well-being have mainly focused on between-school tracking, yet the association between between-school tracking and the development of subjective well-being remains unclear. Previous studies have consistently found that students in academic tracks were more engaged in learning, had higher levels of self-esteem, and felt a stronger sense of school belonging than their counterparts in nonacademic tracks (Burger, 2022; Van Houtte et al., 2012; Van Houtte & Stevens, 2009). This evidence implies that students in academic tracks might experience higher levels of subjective well-being than those in nonacademic tracks. One longitudinal study showed that girls who attended an academic track were likely to have a greater increase in life satisfaction than those in a nonacademic track (Salmela-Aro & Tuominen-Soini, 2010). However, another longitudinal study found a negligible association between changes in life satisfaction and academic-related subjective well-being and school tracks over time (Jerrim & Sims, 2019). Longitudinal research to investigate the association between ability grouping and subjective well-being is currently rare. Thus, this study attempted to replicate previous research to provide more evidence to explain the association between ability grouping and subjective well-being.

#### Achievement composition

Achievement composition is also a paramount school contextual factor closely related to ability grouping to influence adolescents’ psychosocial development (Becker & Neumann, 2018; Marsh et al., 2008). Achievement composition is defined as the achievement differences are associated with the composition of a student body, which is obtained by aggregating student-level achievement to form a class or school-level achievement. Variations in achievement composition are associated with the practice of ability grouping, because the selection of different school tracks is based on students’ achievement levels. However, achievement composition is not necessarily limited to the context of ability grouping, because variations between school tracks are naturally influenced by cohorts and neighborhoods.

Achievement composition is generally used to investigate how school-level achievement composition influences individual-level psychosocial outcomes. Previous literature has found that achievement composition negatively predicted individual-level psychosocial outcomes, particularly academic self-belief (Becker & Neumann, 2018; Frenzel et al., 2007; Marsh et al., 2008). This effect is known as the Big-Fish-Little-Pond effect, which can be viewed as the sum of contrast (negative) and assimilation (positive) effects. The contrast effect refers to situations in which students have more negative perceptions of themselves in high-ability groups than in low-ability groups through an upward social comparison process. The assimilation effect refers to situations in which students are likely to have individual-level behaviors, cognition, and emotions that match the group-level norm through the assimilation process (Cialdini et al., 1976; Kelly, 2009). If the contrast effect is larger than the assimilation effect, the effect is negative, suggesting a Big-Fish-Little-Pond effect exists. Previous studies have found that the Big-Fish-Little-Pond effect affected academic-specific constructs; it is unclear whether the Big-Fish-Little-Pond effect can be observed in general and nonacademic constructs.

When connecting the Big-Fish-Little-Pond effect and the construct of subjective well-being, one might assume that adolescents in academic tracks apply the upward social comparison to form their judgment of subjective well-being. If the upward social comparison (contrast effect) is stronger than the assimilation process (assimilation effect), adolescents in academic tracks should be less satisfied with their general and school lives. However, previous cross-sectional studies focusing on life satisfaction or related constructs have yielded inconsistent results (De Fraine et al., 2002; Rathmann et al., 2018). One cross-sectional study showed that achievement composition positively predicted the perception of general subjective well-being (De Fraine et al., 2002). In contrast, a recent cross-sectional study found no association between achievement composition and life satisfaction (Rathmann et al., 2018). One might argue that life satisfaction is a broader construct that might be less sensitive in capturing the narrower and domain-specific Big-Fish-Little-Pond effect. As the concept of specificity-matching principles was mentioned earlier (Swann et al., 2007), this may explain how the Big-Fish-Little-Pond effect might be more discernible in school satisfaction due to higher levels of content specificity. However, one systematic review demonstrated mixed findings regarding the association between school-related subjective well-being and achievement composition in cross-sectional studies (Belfi et al., 2012).

Based on the aforementioned theoretical considerations and empirical findings, there is inconclusive evidence on how ability grouping and academic composition are associated with the development of subjective well-being. Moreover, achievement composition and institutional differences related to ability grouping are inherently confounded because the varying ability levels of different ability groups lead to diverse curricula and the inherent goal of tracking practices (Duflo et al., 2011; Schofield, 2010). To date, no longitudinal study has systematically investigated how these two contextual factors are associated with the development of subjective well-being. Thus, it appears necessary to explore the distinct relevance of ability grouping and achievement composition in the development of subjective well-being.

### Educational System in Germany

Germany is an appealing case for studying the development of subjective well-being across low- and high-ability groups because the practice of ability grouping is an explicit and rigid between-school tracking. As a result, significant differences in social and learning environments may influence students’ perceptions of their subjective well-being. Although the practice of ability grouping varies in terms of the grade level at which students enter, selection criteria, and the number of ability groups across the 16 federal states (Becker et al., 2016), students are generally sorted into different tracks at the end of primary school (i.e., grade 4 or 6). Formerly, three ability tracks existed: Hauptschule, Realschule, and Gymnasium, corresponding to vocational, intermediate, and academic tracks consisting of low-, middle-, and high-achievement students, respectively. Nowadays, this three-tier system barely exists; the number and type of school tracks vary strongly between German states. However, academic-track schools still exist in all states (Gymnasium; see Becker et al., 2016). The academic track mainly prepares students to enter university by providing an academically oriented curriculum and challenging instruction (Baumert et al., 2010; Henschel et al., 2019). Given that the features of tracks have become more diverse in Germany, other nonacademic track schools can also provide university entrance certificates to students (e.g., comprehensive schools). However, the main aims of these nonacademic tracks are less focused on providing university entrance certificates for students. They primarily offer vocational or intermediate school-leaving certificates to prepare and qualify students for vocational training. Thus, nonacademic tracks provide less demanding curricula and instruction. Consequently, these school tracks are geared toward students with comparatively lower achievement levels.

Thus, between-school tracking creates different academic learning environments. These environments exhibit contrasting achievement compositions related to instruction and curriculum, irrespective of the specific tracks that exist in different states. Therefore, these school tracks can be conceptualized as “differential learning environments,” providing a suitable situation for studying the extent to which school environments affect subjective well-being development during secondary schooling.

### The Selection of Covariates

Gender, immigrant status, socioeconomic status (SES), and prior academic achievement before the transition to secondary school are relevant covariates because they are associated with subjective well-being, ability grouping, and achievement composition. Gender differences in maintaining peer relationship styles, coping with stress, positive and negative affect, and support provisions have been suggested (Rose & Rudolph, 2006 for review), which might lead to different perceptions of subjective well-being. As immigrant adolescents might struggle with large gaps in attitudes, values, and behaviors between their own culture and the host culture (Lui, 2015), they are likely to encounter more stressful life and school experiences. As a result, they may be less satisfied with their lives than non-immigrant children and adolescents. Adolescents with lower SES are likely to face a wide range of stressful events due to insufficient materials and cultural and social assets, which may be associated with poor emotional and social development (Somerville & Whitebread, 2019). With respect to ability grouping, girls, adolescents with higher SES, and non-immigrant adolescents are likely to have better prior academic achievement, resulting in a high likelihood of entering an academic track and different variations in achievement compositions (Hannover & Kessels, 2011; Maaz et al., 2008; Schofield, 2010). As prior academic achievement before the transition to secondary school is an important indicator in deciding the selection of tracks and variations in achievement composition, prior academic achievement was included as a covariate in analyses.

## The Current Study

**Fig. 1:**
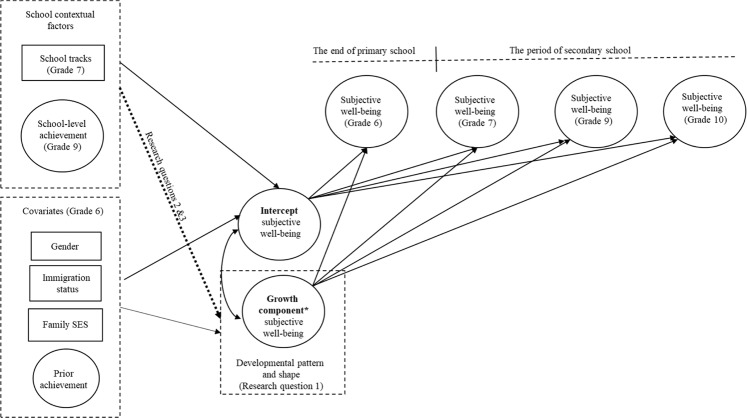
Conceptual model depicting associations between the developmental trends of subjective well-being. Subjective well-being represents life satisfaction or school satisfaction. The circle represents a latent construct. School tracks represent ability grouping; school-level achievement represents achievement composition. *Growth component could be linear slope, quadratic or cubic component

Stage-Environment-Fit theory and empirical research suggested that a broad range of psychosocial variables declined during secondary school. Even though a decline in life satisfaction was found during secondary school, previous studies have paid less attention to school satisfaction. Little is known about whether a decline in subjective well-being is associated with the transition to secondary school. Moreover, no study has considered investigating the longitudinal associations of two important school contextual factors—ability grouping and achievement composition—to investigate their longitudinal associations with subjective well-being. To address the research gap, this study aimed to investigate the development of life satisfaction and school satisfaction and how ability grouping and achievement composition were associated with the development of life satisfaction and school satisfaction (for conceptual figure, see Fig. 1). The first question examined the development of life satisfaction and school satisfaction in terms of overall patterns and shapes. It was expected that all students would experience a decline in life satisfaction and school satisfaction, but the developmental shapes of life satisfaction and school satisfaction would be different. The second question examined the association between ability grouping and the development of life and school satisfaction. The third research question investigated the association between achievement composition and the development of life and school satisfaction. As there was insufficient evidence to set hypotheses for the second and third research questions, these two research questions were exploratory. To gain more confidence in interpreting the relationship between ability grouping and the development of life satisfaction and school satisfaction, gender, immigrant status, SES, and prior achievement before the transition were considered relevant covariates in the analyses.

## Method

### Data

A representative sample from a multi-cohort longitudinal large-scale study in the state of Berlin in Germany was used. The BERLIN-study is a joint project by the Max-Planck-Institute for Human Development (MPIB, Berlin, Germany, Principal Investigator: Prof. Dr. Jürgen Baumert), the DIPF | Leibniz Institute for Research and Information in Education (Frankfurt am Main/Berlin, Germany, Principal Investigator: Prof. Dr. Kai Maaz), and the Leibniz Institute for Science and Mathematics Education (IPN, Kiel, Germany, Principal Investigator: Prof. Dr. Olaf Köller). Students were eligible to participate during the entire lower secondary school period (i.e., grades 6 to 10). In Berlin, between-school tracking is implemented after students complete grade 6 in primary school. Subsequently, students are tracked into two types of public schools: academic track (Gymnasium) and nonacademic track (Integrierte Sekundarschulen, or comprehensive schools). Like other German states, the academic track in Berlin is intended to prepare students for university entrance. While the nonacademic track prepares students for vocational, mid-level, and university entrance certificates, university entrance preparation caters to only a small proportion of students (Baumert et al., 2017). The differences in achievement and other relevant characteristics between academic and nonacademic tracks align accordingly (Becker et al., 2016; Henschel et al., 2019).

The data collection was part of a large-scale study comprising two longitudinal data collections: (a) the first cohort from grades 6 to 7, capturing the transition from primary to lower secondary school (C1 cohort), and (b) the second cohort from lower secondary education in grades 9 to 10 (C2 cohort). Each cohort was sampled by using a two-stage sampling design. In the C1 cohort, public primary schools were randomly sampled, and two classes were randomly sampled from each school. In the C2 cohort, after three years, secondary schools were randomly sampled based on stratification of tracks. Within each school, twenty-five 15-year-old students and ten students who were not yet 15 years old in grade 9 were randomly sampled. This sampling procedure is equivalent to the German PISA sampling rationale (Becker et al., 2017 for further details).

The dataset was designed such that students in the C1 cohort were in grade 9 of secondary school, which is the same grade at which C2 cohort was assessed. In the process of assessing C2 cohort, all students who attended secondary school in the C1 cohort were randomly sampled in the C2 cohort. Schools in the C2 cohort comprised one-third of all secondary schools in Berlin. A third of the original sample in the C1 cohort was eligible for further longitudinal assessments, along with the students in the C2 cohort. Therefore, the longitudinal students in our sample differ neither from the random sample of primary school students in the C1 cohort nor from the random sample in the C2 cohort (for further details, see Becker & Neumann, 2018).

The sample of students followed longitudinally was *N* = 1,841. Data from four time points during grades 6 to 10 were used. The first measurement point was at the end of primary school in grade 6 (April 2011; Time 1). The average age of the students at Time 1 was 12.20 years (*SD* = 0.81). The second measurement was performed immediately after the transition to lower secondary school in grade 7 (October 2011; Time 2). The third measurement was at the end of grade 9 (May/June 2014; Time 3). The final measurement was at the end of grade 10, before the transition to upper secondary or vocational education (May/June 2015; Time 4).

### Measures

#### Life satisfaction (Times 1–4)

A German adaptation of the Temporal Satisfaction with Life scale was used to measure life satisfaction (Trautwein, 2004; sample item: “I am satisfied with my life”). Four 4-point Likert-type items (*1* = *completely disagree; 4* = *completely agree*) measured life satisfaction across the four time points. Internal consistency was good at the four measurement points (*M* = 0.90, *SD* = 0.004). Supplementary Table S1 provides a description of the items for life satisfaction in the Supplemental Materials.

#### School satisfaction (Times 1–4)

Students were asked about their satisfaction with their school life (Bos & Pietsch, 2006; Kelly, 2003; sample item: “I like to go to school”). Four 4-point Likert-type items *(1* = *completely disagree; 4* = *completely agree)* measured school satisfaction. Internal consistency was good at all four measurement points (*M* = 0.84, *SD* = 0.02). Supplementary Table S1 provides a description of the items for school satisfaction in the Supplemental Materials.

#### School track (Time 2)

School track is used as a proxy for ability grouping. The school track included the academic track (Gymnasium) and nonacademic track (Integrierte Sekundarschule). The academic track was coded as 1 and nonacademic track was coded as 0.

#### School-level achievement (Time 3)

School-level achievement represented the achievement composition. School-level achievement was modeled as a latent construct that was composited by individual math and reading achievement in grade 9. All items in the reading and mathematics tests were constructed using items from the 2006 German PISA study (Prenzel et al., 2007, 2008). The math and reading tests followed a multi-matrix design; that is, students randomly received different booklets, each containing different or similar items. Both tests were scaled using the Rasch and partial-credit models (PCM) using ConQuest (Wu et al., 2007). To obtain unbiased estimates of students’ achievement based on the multi-matrix design, five plausible values (PV) were produced for each subject. The reliability was good for reading (*r*_eap_ = 0.90) and mathematics (*r*_eap_ = 0.89). This study included five school-level PVs.

### Covariates

#### Gender (Time 1)

Gender was coded as 0 for boys and 1 for girls.

#### Immigrant status (Time 1)

Students without immigrant backgrounds were coded as 0 and those with at least one parent born outside Germany were coded as 1.

#### Family socioeconomic status (Time 1)

The International Socio-Economic Index of Occupational Status (HISEI; Ganzeboom & Treiman, 2003) was used to represent family socioeconomic status (family SES). HISEI is based on parents’ occupations.

#### Prior achievement (Time 1)

Individual-level achievement in grade 6 represented prior achievement before entering secondary school. Individual prior achievement was modeled as a latent construct composed of individual-level math and reading in grade 6. All items in the reading and math tests were constructed using items directly from the Progress in International Reading Literacy Study (PIRLS; Bos et al., 2003) and the longitudinal state assessment conducted by the state of Hamburg (LAU; Study of Learning Prerequisites; Lehmann & Lenkeit, 2008; Lehmann et al., 2011). The tests were scaled using the Rasch model and PCM. Warm’s likelihood estimate (WLE; Warm, 1989) was applied to obtain students’ reading and math scores because all students took the same reading and math test in grade 6, which suffices for unbiased WLE estimation. Reliability was good for mathematics (*r*_wl*e*_ = 0.91) and reading (*r*_wle_ = 0.85).

### Statistical Approach

#### Measurement invariance (MI)

Measurement invariance must be ensured to examine developmental patterns and shapes of subjective well-being across time and ability tracks. The assumption of measurement invariance for life satisfaction and school satisfaction constructs were examined in the overall group and a multiple-group analyses (i.e., academic vs. nonacademic tracks). When the assumption of scalar invariance is valid, interpretations of mean-level changes across time points, between groups, and within groups across time are meaningful (Little, 2013). The model fit was evaluated using the comparative fit index (CFI), Tucker–Lewis index (TLI), root mean square error of approximation (RMSEA), and standardized root mean square residual (SRMR). Acceptable fit was defined as CFI and TLI ≥ 0.90, and RMSEA and SRMR ≤ 0.08 (Hu & Bentler, 1999; Little, 2013). Given that the chi-square test is likely to be significant due to the large sample size, the chi-square test was not considered to assess model fit for MI. Following recommendations in a methodological study (Chen, 2007), changes in CFI, RMSEA, and SRMR were also taken into account to assess model fit. For testing metric invariance, a change of CFI ≤ | 0.01| that combines with a change of RSMEA ≤ | 0.015| or a change of SRMR ≤ | 0.030| is considered acceptable. For testing scalar invariance, a change of CFI ≤ | 0.010| that combine with a change of RSMEA ≤ | 0.015| or a change of SRMR ≤ | 0.010| is acceptable.

#### Unconditional latent growth model

To examine the developmental patterns and shapes of life satisfaction and school satisfaction (Research Question 1), the latent growth model (LGM) was applied to the overall group (Model 0; baseline model). The LGM as a curves-as-factor model was estimated using the latent factors in the measurement model testing (Duncan et al., 2006). Three LGM models were considered to specify the linear and non-linear growth components: (1) linear, (2) quadratic (non-linear), and (3) cubic (non-linear). In the linear model, the loadings of the latent intercept were fixed as 1 across four occasions, and the loadings for the linear change were fixed as 0, 0.3, 3, and 4 according to the time of measurement (i.e., the interval between T1 and T2 was four months; the interval between T2 and T3 was 32 months, and the interval between T3 and T4 was 12 months). The correlation between the latent intercept and the latent growth component was correlated to understand whether the starting point of a construct was related to the level of change. In the quadratic model, the loadings of the quadratic component were 0, 0.09, 9, and 16 according to the timing of the assessments. In the cubic model, the loadings were set as 0, 0.081, 81, and 256. To assess the model fit, the above cut-off values of the fit indices were used for evaluation. Additionally, the Akaike information criterion (AIC; Akaike, 1987) and Bayesian information criterion (BIC; Schwarz, 1978) were used to select the final model (Kim et al., 2016). Models with smaller AIC and BIC values suggest a better fit.

#### LGM with the effects of school tracks and school-level achievement

After determining the developmental patterns and shapes of life satisfaction and school satisfaction, the effects of school tracks and school-level achievement on growth components of life satisfaction and school satisfaction were investigated (Research Questions 2 and 3). Three models were used to distinguish the effect of school tracks from that of achievement composition. First, the LGM included only school tracks (Model 1), testing the overall effect of school tracks on growth components. School-level achievement was then included to examine the effect of achievement composition by predicting the intercept and growth components in the LGM (Model 2). The final model (Model 3) included individual-level covariates to examine the net context effects for predicting the intercept and growth components.

#### Missing data

Most students had data on either life or school satisfaction at all four measurement time points. Considering that students changed schools from the end of primary school to lower secondary school and that four years of data collection is a long period, the proportion of missing data might not be substantial. Students who did not have life satisfaction data at one measurement point or more (37%) were significantly likely to be immigrant students (*χ*^*2*^(1) = 33.55, *p* < 0.001), have lower levels of family SES (*t* (833.96) = 2.750, *p* < 0.05), prior reading achievement (*t*(1082.50) = 7.751, *p* < 0.001), prior math achievement (*t*(1249.20) = 7.78, *p* < 0.05), or lower life satisfaction at Time 1 (*t*(823.69) = 1.99, *p* < 0.05). Students who did not have school satisfaction data at one measurement point or more (35%) were significantly likely to be immigrants (*χ*^*2*^(1) = 26.33, *p* < 0.001), have lower levels of family SES (*t*(716.48) = 2.43, *p* < 0.05), prior reading achievement (*t*(972.68) = 8.358, *p* < 0.001), prior math achievement (*t*(1127.4) = 8.256, *p* < 0.05), lower school satisfaction at Time 1 (*t*(855.4) = 3.52, *p* < 0.05), Time 2 (*t*(994.51) = 2.45, *p* < 0.05), or Time 3(*t*(565.24) = 3.4, *p* < 0.05). According to the above attrition patterns, the analyses implied that missing patterns are not completely missing at random (MCAR) but at least missing at random (MAR). This makes conventional methods, such as listwise or casewise deletion, inadequate, as they would require the assumption of MCAR to provide unbiased estimates. Following the assumption of MAR, the full information maximum likelihood (FIML) method is a more adequate method for dealing with missing data in this study (Enders, 2010). In addition, FIML includes all available data and works overall, similar to other inclusive strategies, such as multiple imputation (Enders, 2010). Hence, FIML was applied to deal with the missing data in the analyses.

All analyses were conducted in Mplus 8.6 (Muthén & Muthén, 2017) based on maximum likelihood estimation with robust standard errors (MLR). A complex regression approach (Mplus’s analysis option TYPE = COMPLEX) to account for the hierarchical data structure was applied, but it did not differentiate the variance components between levels. School level and individual achievement were standardized before the analyses. As mentioned above, the FIML approach in Mplus was used to handle the missing data. Sampling weights were also included to account for the two-stage sampling.

## Results

### Descriptive Statistics and Correlations

The means and standard deviations for the study variables for the overall group, academic track, and nonacademic track are presented in Table 1. Table 1 shows that life satisfaction and school satisfaction generally decreased over time in both tracks, yet school satisfaction increased from grade 6 (Time 1) to grade 7 (Time 2). The trajectories of life satisfaction between the academic and nonacademic tracks converged in grade 10 (Time 4). Girls, students with higher family SES, German students, or students with higher math and reading achievement in grade 6 were likely to be in the academic track. The academic track showed a higher level of school-level math and reading achievement.

**Table 1:** Descriptive statistics for study variables

	Overall		Academic track		Non-academic track	
	*M*/%	*SD*	*M*/%	*SD*	*M*/%	*SD*
Girl (T1)	0.484	−	0.51	−	0.47	−
Family SES (T1)	51.13	20.97	58.43	20.35	44.39	19.21
Immigrants (T1)	0.45	−	0.39	−	0.50	−
Academic track (T2)	0.43	−	−	−	−	−
Prior reading achievement^a^ (T1)	1.06	0.98	1.57	0.87	0.68	0.88
Prior math achievement^a^ (T1)	0.14	1.16	0.76	1.12	−0.33	0.95
School-level reading achievement^b^ (T3)	0.10	0.91	0.80	0.46	−0.43	0.79
School-level math achievement^b^(T3)	−0.77	0.94	−0.04	0.62	−1.31	0.74
Life satisfaction (T1)	3.44	0.70	3.52	0.64	3.38	0.74
Life satisfaction (T2)	3.36	0.71	3.40	0.65	3.33	0.76
Life satisfaction (T3)	3.18	0.73	3.19	0.69	3.18	0.77
Life satisfaction (T4)	3.13	0.74	3.13	0.73	3.13	0.75
School satisfaction (T1)	3.04	0.74	3.18	0.68	2.93	0.77
School satisfaction (T2)	3.27	0.63	3.39	0.50	3.18	0.70
School satisfaction (T3)	2.64	0.72	2.76	0.70	2.54	0.71
School satisfaction (T4)	2.57	0.78	2.65	0.74	2.51	0.80

^a^Descriptive statistics of prior math and reading achievement for individuals were presented because the latent constructs of general prior achievement was defaulted as mean 0 and standard deviation as 1

^b^Descriptive statistics of school-level math and reading achievement were presented because the latent construct of general school-level achievement was defaulted as mean 0 and standard deviation as 1

Table 2 presents the correlations among the study variables. The latent construct of school-level achievement was significantly positively related to school satisfaction over time, yet school-level achievement was only significantly positively related to life satisfaction at Times 1 and 2. Female gender, family SES, academic track, and the latent of prior achievement were significantly positively related to school satisfaction at most time points. Life satisfaction was significantly positively related to school satisfaction over time.

**Table 2:** Correlations for study variables

	1	2	3	4	5	6	7	8	9	10	11	12	13	14
1. Girls		−0.052	−0.019	0.037	−0.031	0.024	−0.005	−0.085**	−0.129***	−0.091**	0.202***	0.076**	0.078**	0.070*
2. Family SES			−0.366***	0.346***	0.581***	0.468***	0.023	0.036	−0.033	0.014	0.082**	0.124***	0.078*	0.044
3. Immigrants				−0.115***	−0.465***	−0.342***	0.012	−0.033	−0.002	0.043	−0.019	−0.100***	−0.093**	0.011
4. Academic track					0.557***	0.712***	0.111***	0.044	0.012	−0.001	0.169***	0.166***	0.152***	0.105***
5. Prior performance^a^ (T1)						0.740***	0.030	0.023	−0.031	−0.018	0.170***	0.179***	0.148***	0.065*
6. School-level performance^b^ (T 3)							0.112***	0.063*	−0.005	−0.018	0.209***	0.250***	0.206***	0.092**
7. Life satisfaction (T1)								0.509***	0.311***	0.309***	0.383***	0.276***	0.235***	0.209***
8. Life satisfaction (T2)									0.359***	0.330***	0.251***	0.401***	0.245***	0.202***
9. Life satisfaction (T3)										0.562***	0.130***	0.146***	0.295***	0.306***
10. Life satisfaction (T4)											0.145***	0.127***	0.243***	0.343***
11.School satisfaction (T1)												0.388***	0.334***	0.280***
12. School satisfaction (T2)													0.358***	0.244***
13. School satisfaction (T3)														0.613***
14. School satisfaction (T4)														

^a^The latent constructs of general prior achievement for individuals was composited by prior math and reading achievement for individuals

^b^The latent construct of general school-level achievement was composited by prior math and reading achievement for individuals

*p < 0.05; **p < 0.01; ***p < 0.001

### Measurement Invariance for the Overall Group and Multiple-group Analyses across Time

The first step involved testing the assumption of measurement invariance for the two well-being constructs for the overall group and within subgroups (i.e., academic vs. nonacademic track) across the four time points. For the overall and multiple-group models, fit indices showed a good fit for the life satisfaction and school satisfaction constructs (see Table 3). Regarding school satisfaction, fit indices were acceptable. Altogether, the assumption of scalar invariance was valid for life satisfaction and school satisfaction in the overall group and the multiple-group analyses. Thus, it was meaningful to compare the means and mean-level changes across time within and between school tracks.

**Table 3:** Test for measurement invariance over the four time points for the overall group and multiple-group models (academic and nonacademic tracks)

Grouping	Construct	Invariance test	RMSEA	CFL	TLI	SRMR
Overall	Life satisfaction	Configural	0.020	0.995	0.992	0.022
		Metric	0.020	0.995	0.992	0.028
		Scalar	0.023	0.992	0.989	0.033
	School satisfaction	Configural	0.053	0.957	0.930	0.033
		Metric	0.052	0.953	0.932	0.042
		Scalar	0.050	0.952	0.937	0.042
Multigroup^a^	Life satisfaction	Configural	0.026	0.993	0.988	0.027
		Metric	0.025	0.992	0.988	0.054
		Scalar	0.029	0.988	0.985	0.057
	School satisfaction	Configural	0.055	0.952	0.923	0.037
		Metric	0.053	0.949	0.927	0.054
		Scalar	0.057	0.934	0.917	0.071

^a^Academic track vs. nonacademic track

### Development of Life Satisfaction and School Satisfaction across Time

To test the developmental patterns and shapes of life satisfaction and school satisfaction, linear and nonlinear LGM models in the overall group and for academic and nonacademic tracks were conducted separately. The complexity of the models was tested using a stepwise procedure by adding parameters from models with only a linear component to nonlinear models with quadratic and cubic components. For life satisfaction, fit indices indicated a good fit for both the overall and academic and nonacademic track analyses for the linear, quadratic, and cubic models (see Supplementary Table S2). AIC tended to select the less parsimonious models (i.e., quadratic or cubic models) in most analyses; however, BIC tended to favor the more parsimonious model (i.e., linear model) in most analyses. Given that the complex models did not lead to large changes in the AIC and BIC values and fit indices, the development of life satisfaction was characterized by a linear shape (Model 0).

School satisfaction showed a different developmental shape. Supplementary Table S2 shows that the less parsimonious models performed progressively better (i.e., quadratic and cubic models; see Supplementary Table S2 in the Supplemental Materials, lower panel). Absolute model fit was clearly the best in the cubic model for both the overall group and the multiple-group analyses for each group. In this specification, all fit indices were acceptable, with only SRMR indicating a slight misfit for the academic group. Moreover, based on the AIC and BIC values, the results consistently favored the cubic model in all analyses of school satisfaction. Thus, the development of school satisfaction was characterized by a non-linear shape (Model 0).

**Fig. 2:**
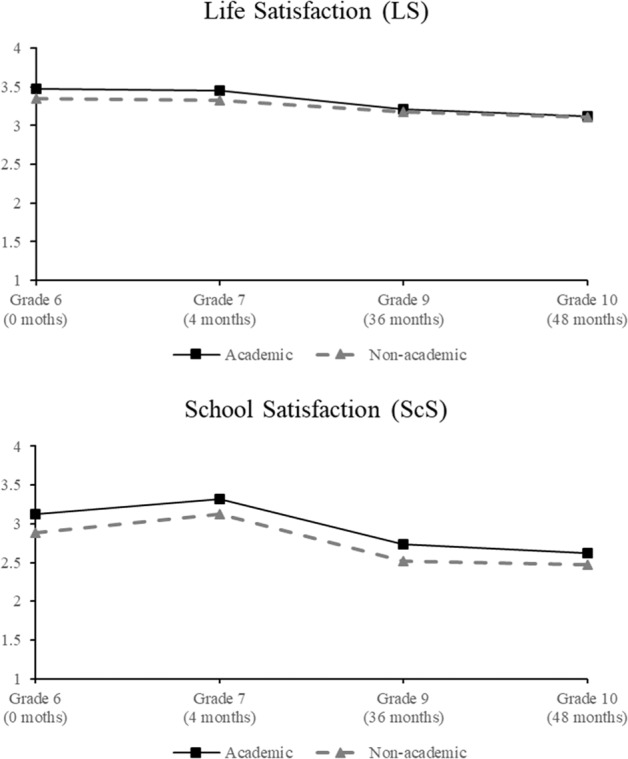
Estimated latent mean-changes in life satisfaction and school satisfaction in academic and nonacademic tracks

Table 4 shows the parameter estimates for the final baseline models (Model 0) for life and school satisfaction for the overall group analyses. For life satisfaction, the results demonstrated that the linear slope was significantly negative, suggesting that life satisfaction declined linearly over time. However, school satisfaction exhibited a non-linear relationship. Significant positive linear and negative quadratic components represent the rise after the transition and fall in the long run across secondary schooling. The cubic component yielded statistical significance, indicating a diminishing decrease in development toward grade 10. Figure 2 shows the trajectories of life and school satisfaction for both tracks. Overall, life satisfaction and school satisfaction exhibited similar developmental patterns after entering secondary school; that is, both decreased over time. In addition, students in the academic track generally had higher levels of life and school satisfaction.

**Table 4:** Parameters estimates of the baseline models (Model 0) for life satisfaction and school satisfaction in the overall group

	Life satisfaction		School satisfaction	
*Construct*	Parameter	*SE*	Parameter	*SE*
Latent mean				
Intercept	3.402***	0.020	2.984***	0.028
Linear slope	−0.072***	0.006	0.859***	0.087
Quadratic			−0.438***	0.036
Cubic			0.012***	0.001
Latent variance				
Intercept	0.269***	0.019	0.160***	0.016
Linear slope	0.019***	0.002	0.015***	0.002
Covariance				
Intercept with linear slope	−0.028***	0.005	−0.013**	0.004

*p < 0.05; **p < 0.01; *** *p* < 0.001

### The Effects of Ability Grouping and Achievement Composition on the Development of Life Satisfaction and School Satisfaction

To answer the research questions about how the effects of school tracks and school-level achievement were associated with the development of life satisfaction and school satisfaction, overall group LGM analyses were examined. The baseline model (Model 0) was extended to three sequential models, probing for (a) the effect of tracks on a growth component (Model 1), (b) the effect of school-level achievement on growth components (Model 2), and (c) the extent to which the effects of school tracks and school-level achievement on a growth component hold after controlling for individual-level covariates (Model 3). The interpretation of the results mainly relies on Model 3, as it was the final model. To interpret the effects of school tracks and school-level achievement on the development of life satisfaction and school satisfaction, the main focus lies on the predictions of school tracks and school-level achievement on growth components. Regarding the effects of school tracks and school-level achievement on life satisfaction and school satisfaction before the transition to secondary school (i.e., grade 6), they only provided information about how the initial levels of life satisfaction and school satisfaction were related to the later assignment of school tracks and variations in school-level achievement composition. The results for life satisfaction and school satisfaction were presented successively.

Table 5 shows the predictions of school tracks and school-level achievements on life satisfaction in the overall group analyses. Model 1 confirmed that the initial level of life satisfaction was significantly higher in the academic track than in the nonacademic track (*β* = 0.127, *p* < 0.05), and the linear slope was more significantly negative for the academic track than for the nonacademic track (*β* = −0.030, *p* < 0.05). Model 2 showed a significant positive effect of school-level achievement on the intercept (*β* = 0.053, *p* < 0.05) and a significant negative effect on the linear slope (*β* = −0.019, *p* < 0.05). However, the effect of school track on the linear slope was statistically insignificant in Model 2. When individual-level covariates were controlled for in Model 3, there were no substantive changes in any estimates or the results of significant tests. In Model 3, academic track and school-level achievement were significantly and positively related to the intercept. Although the intercept was not the main focus of this study, the results suggested that students who had higher initial levels of life satisfaction at the end of primary school were likely to attend an academic track or secondary school with higher levels of achievement composition. Only school-level achievement was significantly negatively related to the linear slope (*β* = −0.035, *p* < 0.05), suggesting that students in schools with higher levels of achievement composition were likely to experience a greater decline in life satisfaction.

**Table 5:** Unstandardized estimated parameters of LGM for life satisfaction predicted by academic track, school-level performance and individual-level covariates in the overall group

	Model 1		Model 2		Model 3	
	LGM + track		LGM + track+ school-level performance		LGM + track+ school-level performance +covariates	
*Construct*	Parameter	*SE*	Parameter	*SE*	Parameter	*SE*
Latent mean						
Intercept	3.347***	0.023	3.377***	0.025	3.422***	0.064
Linear slope	−0.059***	0.008	−0.070***	0.009	−0.063**	0.024
Latent variance						
Intercept	0.265***	0.019	0.263***	0.019	0.239***	0.021
Linear slope	0.019***	0.002	0.019***	0.002	0.018***	0.002
Intercept predicted by						
Girl (0 = boy)					−0.101*	0.036
Family SES					0.000	0.001
Immigrant (0 = no immigrant)					0.011	0.045
Prior performance					−0.073	0.043
Academic track (0 = non-acad.)	0.127***	0.036	0.055	0.044	0.106*	0.054
School-level achievement			0.053*	0.024	0.087*	0.039
Linear slope predicted by						
Girl (0 = boy)					−0.026	0.014
Family SES					0.000	0.000
Immigrant (0 = no immigrant)					−0.003	0.014
Prior achievement					0.022	0.017
Academic track (0 = non-acad.)	−0.030**	0.012	−0.004	0.014	−0.002	0.016
School-level achievement			−0.019**	0.007	−0.035*	0.014
Covariance						
Intercept with linear slope	−0.027***	0.005	−0.027***	0.005	−0.027***	0.006

Individual math

*Prior achievement* individual-level achievement in grade 6, *School-level achievement* school-level achievement in grade 9, *Nonacad.* nonacademic track

*p < 0.05; **p < 0.01; ***p < 0.001

Table 6 presents the results of the effects of school track and achievement composition on the developmental components of school satisfaction. Model 1 showed that academic track significantly positively predicted the initial levels of school satisfaction (*β* = 0.238, *p* < 0.05). The prediction of school track on the linear slope, quadratic, and quadratic components was statistically insignificant. In Model 2, school-level achievement significantly positively predicted only the intercept. None of the growth components were significantly predicted by school tracks or school-level achievement in Model 2. As individual-level covariates were included in Model 3, the estimated parameters and significant tests remained similar. In Model 3, only school-level achievement significantly positively predicted the intercept, suggesting that students with higher initial levels of school satisfaction at the end of primary school were likely to attend secondary schools with higher levels of achievement composition. Neither school track nor school-level achievement significantly predicted any growth components.

**Table 6:** Unstandardized estimated parameters of LGM for school satisfaction predicted by academic track, school-level performance and individual-level covariates in the overall group

	Model 1		Model 2		Model 3	
	LGM + track		LGM + track+ school-level performance		LGM + track+ school-level performance +covariates	
*Construct*	Parameter	*SE*	Parameter	*SE*	Parameter	*SE*
Latent mean						
Intercept	2.881***	0.028	2.956***	0.030	2.849***	0.064
Linear slope	0.922***	0.119	0.996***	0.124	1.151***	0.170
Quadratic	−0.465***	0.050	−0.493***	0.052	−0.525***	0.063
Cubic	0.013***	0.001	0.013***	0.001	0.013***	0.002
Latent variance						
Intercept	0.148***	0.015	0.136***	0.013	0.116***	0.015
Linear slope	0.015***	0.002	0.015***	0.002	0.018***	0.002
Intercept predicted by						
Girl (0 = boy)					0.172***	0.036
Family SES					0.001	0.001
Immigrant (0 = no immigrant)					0.092*	0.036
Prior achievement					0.003	0.039
Academic track (0 = nonacad.)	0.238***	0.050	0.060	0.058	0.032	0.059
School-level achievement			0.130***	0.030	0.164***	0.038
Linear slope predicted by						
Girl (0 = boy)					−0.115*	0.052
Family SES					−0.002	0.001
Immigrant (0 = no immigrant)					−0.195***	0.055
Prior achievement					−0.026	0.047
Academic track (0 = nonacad.)	−0.148	0.181	−0.330	0.242	−0.242	0.232
School-level achievement			0.137	0.112	−0.074	0.118
Quadratic component						
Girl (0 = boy)					0.027*	0.013
Family SES					0.000	0.000
Immigrant (0 = no immigrant)					0.048***	0.014
Prior achievement					0.009	0.012
Academic track (0 = nonacad.)	0.065	0.076	0.131	0.102	0.119	0.099
School-level achievement			−0.050	0.048	0.029	0.050
Cubic component						
Academic track	−0.002	0.002	−0.003	0.003	−0.003	0.003
School-level achievement			0.001	0.001	−0.001	0.001
Covariance						
Intercept with linear slope	−0.012*	0.004	−0.010**	0.003	−0.012**	0.004

*Prior achievement* individual-level achievement in grade 6, *School-level achievement* school-level achievement in grade 9, *Nonacad.* nonacademic track

*p < 0.05; **p < 0.01; ***p < 0.001

### Sensitivity Analyses

As the overall group analyses may not fully detect differences between school tracks, multiple-group LGM models were conducted to examine whether the results were consistent with the LGM model in the overall group analyses. In the multiple-group LGM models, more parameters would be estimated because every parameter could vary between academic and nonacademic tracks. Parallel to the overall LGM model, three models were conducted: (a) Model 1 without school-level achievement and covariates, (a) Model 2 with the inclusion of school-level achievement, and (b) Model 3 with the inclusion of school-level achievement and covariates. The results of the multiple-group LGM models are provided in the Supplemental Materials.

Multiple-group LGM models of life satisfaction showed significant differences in linear slopes between the academic and nonacademic tracks in Models 1 and 2 (Supplementary Table S3 in Supplemental Materials). When school-level achievement and individual-level covariates were included in Model 3, there were no significant differences in the linear slopes between the academic and nonacademic tracks. In Model 3, school-level achievement did not significantly predict the linear slopes in the academic and nonacademic tracks, suggesting that the effect of school-level achievement was homogenous across schools. The effect of school-level achievement on the linear slope was statistically weaker in the multiple-group LGM models than in the overall LGM models, which might be due to higher parameterization in the multiple-group LGM models. The results of overall and multiple-group LGM models converged on the finding that the effect of school track was not significantly associated with the development of life satisfaction.

Regarding school satisfaction, Model 2 showed that school-level achievement in the nonacademic track significantly predicted the linear slope and quadratic components (Supplementary Table S4 in Supplemental Materials). Once individual-level covariates were included in Model 3, the effect of school-level achievement did not significantly predict the linear slope and quadratic components in the nonacademic track. In addition, the effect of school-level achievement did not differ significantly between school tracks. Only the effect of school-level achievement on the cubic significantly differed between tracks (Δ_Cubic(a-non)_ = −0.004), but differences in the effect were negligible. Both overall and multiple-group LGM models converged on the finding that the effects of school track and school-level achievement were weakly associated with the development of school satisfaction.

## Discussion

Declining patterns in a wide range of psychosocial constructs have been documented during the transition to secondary school. Less is known about whether and to what extent subjective well-being declines during secondary school. Moreover, no longitudinal study has investigated how ability grouping and achievement composition are associated with the development of subjective well-being. This study examined the trajectories of life satisfaction and school satisfaction; the relationships between the trajectories of life satisfaction and school satisfaction and school tracks; and the relationships between the trajectories of life satisfaction, school satisfaction, and school-level achievement. The results show that life and school satisfaction declined over time, yet the developmental shapes of life satisfaction and school satisfaction were different. Moreover, the results reveal that school track is weakly associated with the development of life and school satisfaction. However, school-level achievement composition has a negative impact on the development of life satisfaction.

### The Development of Life Satisfaction and School Satisfaction

Overall, life and school satisfaction gradually declined after the transition to secondary school. These results support the hypothesis of the Stage-Environment-Fit model that environmental changes in secondary schools have a negative impact on adolescent development (Eccles et al., 1993). Furthermore, the result mirrors empirical findings that an environmental misfit hindered adolescents’ academic-specific and general psychosocial development (Engels et al., 2017; Loukas et al., 2016; Scherrer & Preckel, 2019). Thus, the findings highlight that the transition to secondary school negatively influences *not only* psychosocial dimensions *but also* subjective well-being.

Although the general declining patterns of life satisfaction and school satisfaction were approximately similar after entering secondary school, the developmental shapes of life satisfaction and school satisfaction differed. Life satisfaction demonstrated a linear shape, but school satisfaction showed a more complex, non-linear shape. Although this study does not test the bottom-up perspective that the developmental shape of domain-specific satisfaction might diverge from the developmental shape of life satisfaction (Diener, 1984), the distinct developmental shapes of life and school satisfaction are in line with the bottom-up perspective. These results extend previous findings that found that the developmental shape of life satisfaction might not be necessarily similar to other domain-specific satisfaction in adult populations (Easterlin, 2010; McAdams et al., 2012). As this study only focuses on one domain-specific satisfaction, future studies should consider trajectories of other relevant domain-specific satisfaction during adolescence to obtain a more detailed bottom-up perspective in adolescent populations.

Furthermore, the distinct developmental period between life and school satisfaction was located between the end of primary school and immediately after the transition to lower secondary school. During this period, school satisfaction temporarily increased. The potential explanation of the temporal increase in school satisfaction might be associated with the “after-effect of summer vacation” because the data collection at the transition to secondary school was right after the summer vacation (Knoppick et al., 2015). Another related explanation might be that a “honeymoon effect” happens immediately after the change in environment because adolescents might be excited about new school experiences (Booth & Gerard, 2014). This explanation is derived from organizational psychology, in which a temporal increase in job satisfaction immediately after a job change has also found (Boswell et al., 2005). As job and school satisfaction are associated with the main working environment for adults and adolescents, respectively, this explanation might be plausible.

### The Effects of Ability Grouping and Achievement Composition on the Development of Subjective Well-being

School tracks were weakly associated with declines in life and school satisfaction. This might be due to school tracks being associated with many institutional differences in the different dimensions of a school environment (Wang & Degol, 2016). It is possible that the effect of school tracks might be less salient because the positive effects of some dimensions on subjective well-being might compensate for negative effects of other domains within a school track. Future research should consider a multidimensional perspective of the school environment to shed light on the association between ability grouping and subjective well-being. Although a previous study found a positive effect of an academic track on the increasing patterns of life satisfaction (Salmela-Aro & Tuominen-Soini, 2010), the different results might be associated with the different practices of ability grouping. As the empirical evidence is very meager, future studies should investigate this topic in other educational systems to advance knowledge of the longitudinal effect of ability grouping on subjective well-being.

The effect of achievement composition was negatively associated with the decline magnitudes of life satisfaction even after controlling the effects of school tracks and relevant covariates. This robust finding suggests that the Big-Fish-Little-Pond effect is discernible for life satisfaction. This finding is in line with one aspect of environmental changes in the Stage-Environment-Fit model. In the Stage-Environment-Fit model, a secondary school environment provides more demanding learning tasks, has a stricter grading system, and creates a more achievement focused and competitive learning environment (Eccles et al., 1993), which echoes the underlying process of the Big-Fish-Little-Pond effect: students in more academic-oriented environments are likely to experience lower levels of academic self-beliefs (Marsh et al., 2008).

However, achievement composition was not statistically significantly associated with the declining magnitude of school satisfaction, although the direction of the effect of achievement composition on the declining magnitude of school satisfaction was negative. The result is not in line with the specificity-matching principle that a domain-specific variable might be likely to detect a stronger effect of another domain-specific variable due to highly overlapping content (Swann et al., 2007). Moreover, this finding is not in line with the Big-Fish-Little-Pond effect literature, which found that a narrower construct of academic self-concept was more susceptible to environmental changes (Becker & Neumann, 2018). These surprising results might be related to statistical reasons, as the non-linear model of school satisfaction is more complex than the linear model of life satisfaction. To solve the problem of model convergence of the non-linear model, the variances of the quadratic and cubic components were fixed to zero. Furthermore, individual covariates were not included in the prediction of the cubic term, owing to the issue of model non-convergence. Given these complex settings in the school satisfaction model, the statistical power of the effect of achievement composition on the declining magnitude of school satisfaction might be reduced. This insignificant finding indicates that the association between contextual factors and school satisfaction requires further validation.

### Limitations

This study overcomes the limitations of previous studies by including more time points, pre-tracking subjective well-being data, global and domain-specific subjective well-being measures, and two important school contextual factors. However, this study has some limitations. First, it focuses only on between-school tracking. This might limit generalizability to other types of ability grouping (i.e., within schools and course-by-course). Different types of ability grouping might shape more or less heterogeneous environment compared to between-school tracking, which might affect the level of institutional resources and the social comparison process (Chmielewski et al., 2013). Second, given the nature of the data, the period from grades 7 to 9 is much longer than other time points (i.e., grades 6–7 and 9–10). Although this study provides a picture of the short-term changes during the transition between primary and secondary schooling, it is less informative on the specific developmental shape between grades 7 and 9. Future research should investigate how short-term changes are related to long-term changes using data with a more evenly spread measurement points. Relatedly, school satisfaction shows a curvilinear pattern, yet the second turning point (from grades 9 to 10) did not show a clear pattern of whether school satisfaction would increase or remain flat. Studies investigating middle to late adolescence have found an increasing pattern (Salmela-Aro & Tuominen-Soini, 2010; Steinmayr et al., 2019). It would be worthwhile to explore later developmental patterns in upper secondary school. Finally, school-level achievement is used to represent achievement composition as there is limited information on class membership. However, literature on the Big-Fish-Little-Pond effect suggests that class-level achievement composition may exhibit stronger effects than school-level achievement composition (De Fraine et al., 2003; Opdenakker et al., 2002; Opdenakker & Van Damme, 2001). Bearing this limitation in mind, the effect of achievement composition on the development of life satisfaction and school satisfaction may be estimated as the lower limit at the school level, and the effects of the specific classroom composition may be higher.

## Conclusion

Adolescents generally experience environmental misfit during their transition to secondary school, leading to a decline in important psychosocial dimensions. A critical research gap remains regarding the development of subjective well-being and its associations with school contexts. This study investigates the development of life satisfaction and school satisfaction during secondary school, and how two important school contextual factors—ability grouping and achievement composition—are associated with the development of life and school satisfaction. The results underscore that both life satisfaction and school satisfaction decline during secondary school, supporting the Stage-Environment-Fit model (Eccles et al., 1993). The results further highlight that the developmental shape of school satisfaction differed from life satisfaction, which is rather in line with a bottom-up perspective of life satisfaction (Diener, 1984). Particularly, the decline in life satisfaction is associated with achievement composition, underscoring the relevance of achievement performance in secondary school environments for the development of life satisfaction, which is in line with the Stage-Environment-Fit model and the Big-Fish-Little-Pond effect. Taken together, these findings emphasize the difference between the developmental shapes of global and domain-specific subjective well-being and their longitudinal context sensitivity. Future studies should investigate these topics through the lens of a multidimensional perspective of subjective well-being.

## Supplementary Information

Supplemental Materials

## Footnotes

1 We used the terms “ability grouping” and “tracking” interchangeably in this study.
