# Supplementary material for: Association between School Contexts and the Development of Subjective Well-Being during Adolescence: A Context-Sensitive Longitudinal Study of Life Satisfaction and School Satisfaction
Source: J Youth Adolesc. 2023 Jan 19;52(5):1039–57. doi: 10.1007/s10964-022-01727-w (PMC10027814; doi:10.1007/s10964-022-01727-w)
Supplement: Supplementary file 1 — Supplemental Materials [file 10964_2022_1727_MOESM1_ESM.docx]

**Supplemental Materials**

**Table S1** Item Descriptions of Life Satisfaction and School Satisfaction

| Constructs | Items |
| --- | --- |
| Life Satisfaction | I am satisfied with my life. |
|  | I am happy the way that I am living right now. |
|  | I like my life in a way like right now. |
|  | I have everything that is important to me. |
| School Satisfaction | I like going to school. |
|  | I feel comfortable in our school. |
|  | Our school looks friendly. |
|  | Learning is fun in our school. |

**Table S2**

*Model Comparison for the Latent Growth Curve Model (LGM) for Linear and Nonlinear Models in the Overall, Academic, and Nonacademic Groups*

| Construct | Sample | Model | CFI | TLI | RMSEA | SRMR | AIC | BIC |
| --- | --- | --- | --- | --- | --- | --- | --- | --- |
| Life satisfaction^1^ | Overall group | Linear | .991 | .989 | .024 | .048 | 41877.110 | 42163.823 |
|  |  | Quadratic | .991 | .989 | .024 | .044 | 41870.817 | **42163.044** |
|  |  | Cubic | .991 | .989 | .023 | .043 | **41869.296** | 42167.036 |
|  | Academic track | Linear | .986 | .983 | .031 | .056 | 11815.048 | 12037.433 |
|  |  | Quadratic | .987 | .985 | .030 | .054 | 11810.414 | **12037.076** |
|  |  | Cubic | .988 | .985 | .029 | .052 | **11807.454** | 12038.392 |
|  | Nonacademic track | Linear | .989 | .987 | .027 | .053 | **30044.618** | **30313.504** |
|  |  | Quadratic | .989 | .987 | .027 | .050 | 30044.887 | 30318.944 |
|  |  | Cubic | .989 | .986 | .027 | .050 | 30046.783 | 30326.011 |
| School satisfaction^2^ | Overall group | Linear | .924 | .909 | .060 | .098 | 55154.092 | 55440.890 |
|  |  | Quadratic | .924 | .908 | .060 | .098 | 55155.194 | 55447.507 |
|  |  | Cubic | .946 | .934 | .051 | .067 | **54882.352** | **55180.181** |
|  | Academic track | Linear | .892 | .871 | .074 | .149 | 15443.372 | 15665.757 |
|  |  | Quadratic | .893 | .870 | .074 | .149 | 15444.514 | 15671.176 |
|  |  | Cubic | .919 | .901 | .065 | .111 | **15357.473** | **15588.412** |
|  | Nonacademic track | Linear | .917 | .900 | .061 | .084 | 39738.215 | 40007.220 |
|  |  | Quadratic | .917 | .899 | .061 | .084 | 39740.194 | 40014.374 |
|  |  | Cubic | .941 | .928 | .052 | .061 | **39555.954** | **39835.307** |

*Note.* The **bold** values indicate the smallest value across the three models.

^1^To assure model convergence in the cubic model for LS, the variances of the quadratic and cubic components had to be fixed at 0.

^2^To assure model convergence in the quadratic model, the variance of the quadratic component was fixed at 0, and in the cubic model, the variances of the quadratic and cubic components had to be fixed at 0.

**Table S3**

*Unstandardized Estimated Parameters for Life Satisfaction in the Multiple-Group Latent Growth Curve Model in the Academic and Nonacademic Tracks*

|  | Academic track | | | | | |  | Nonacademic track | | | | | |
| --- | --- | --- | --- | --- | --- | --- | --- | --- | --- | --- | --- | --- | --- |
|  | Model 1  (Track) | | Model 2  (+School-level  performance) | | Model 3  (+Covariates) | |  | Model 1  (Track) | | Model 2  (+School-level  performance ) | | Model 3  (+Covariates) | |
| *Construct* | Parameter | *SE* | Parameter | *SE* | Parameter | *SE* |  | Parameter | *SE* | Parameter | *SE* | Parameter | *SE* |
| ***Latent mean*** | | | | | | | | | | | | | |
| Intercept | 3.472*** | .027 | 3.471*** | .027 | 3.552*** | .109 |  | 3.346*** | .021 | 3.345*** | .021 | 3.389*** | .086 |
| Linear slope | -.089*** | .009 | -.089*** | .009 | -.078* | .039 |  | -.058*** | .007 | -.058*** | .007 | -.046 | .028 |
| Δ_Linear slope (a-non)_ | -.031** | .011 | -.031** | .011 | -.032 | .048 |  |  |  |  |  |  |  |
| ***Latent variance*** | | | | | | | | | | | | | |
| Intercept | .180*** | .023 | .173*** | .023 | .157*** | .026 |  | .322*** | .024 | .322*** | .024 | .302*** | .028 |
| Linear slope | .013*** | .003 | .013*** | .003 | .013*** | .003 |  | .022*** | .003 | .022*** | .003 | .022*** | .003 |
| ***Intercept component*** | | | | | | | | | | | | | |
| Girl (0 = boy) |  |  |  |  | -.046 | .057 |  |  |  |  |  | -.150** | .051 |
| Family SES |  |  |  |  | -.001 | .002 |  |  |  |  |  | .001 | .002 |
| Immigrant (0 = no immigrant) |  |  |  |  | .057 | .071 |  |  |  |  |  | -.014 | .054 |
| Prior achievement |  |  |  |  | -.080 | .056 |  |  |  |  |  | -.060 | .056 |
| School-level achievement |  |  | .085** | .028 | .121** | .041 |  |  |  | .013 | .023 | .029 | .040 |
| ***Linear component*** | | | | | | | | | | | | | |
| Girl (0 = boy) |  |  |  |  | -.038 | .019 |  |  |  |  |  | -.016 | .017 |
| Family SES |  |  |  |  | .000 | .001 |  |  |  |  |  | .000 | .001 |
| Immigrant (0 = no immigrant) |  |  |  |  | -.020 | .024 |  |  |  |  |  | .013 | .018 |
| Prior achievement |  |  |  |  | .016 | .022 |  |  |  |  |  | .025 | .020 |
| School-level achievement |  |  | -.019* | .010 | -.029 | .016 |  |  |  | -.012 | .007 | -.023 | .012 |
| Δ_School-level. ach_. _(a-non)_ |  |  | -.007 | .012 | -.006 | .020 |  |  |  |  |  |  |  |
| ***Covariance*** |  |  |  |  |  |  |  |  |  |  |  |  |  |
| Intercept with linear slope | -.008 | .006 | -.006 | .006 | -.007 | .006 |  | -.041*** | .006 | -.040*** | .006 | -.044*** | .008 |

*Note*. Prior performance = individual-level achievement in grade 6. School-level achievement = school-level achievement in grade 9. Nonacad. = nonacademic track. Δ_a-non_ = Difference between academic and nonacademic tracks for the specific parameter (i.e., mean, slope, or regression effect difference, respectively). Ach.= achievement

**p*<.05. ***p*<0.01. *** *p* <.001

**Table S4**

*Unstandardized Estimated Parameters for School Satisfaction in the Nonlinear Multiple-Group Latent Growth Curve Model in the Academic and Nonacademic Tracks*

|  | Academic track | | | | | |  | Nonacademic track | | | | | |
| --- | --- | --- | --- | --- | --- | --- | --- | --- | --- | --- | --- | --- | --- |
|  | Model 1  (Track) | | Model 2  (+School-level math) | | Model 3  (+Covariates) | |  | Model 1  (Track) | | Model 2  (+School-level math) | | Model 3  (+Covariates) | |
|  | Parameter | *SE* | Parameter | *SE* | Parameter | *SE* |  | Parameter | *SE* | Parameter | *SE* | Parameter | *SE* |
| **Latent mean** | | | | | | | | | | | | | |
| Intercept | 3.119*** | .031 | 3.119*** | .031 | 2.975*** | .097 |  | 2.886*** | .025 | 2.885*** | .025 | 2.765*** | .069 |
| Linear slope | .805*** | .111 | .806*** | .111 | .908*** | .195 |  | .918*** | .098 | .905*** | .098 | 1.186*** | .151 |
| Quadratic | -.414*** | .047 | -.414*** | .046 | -.403*** | .063 |  | -.463*** | .041 | -.459*** | .041 | -.547*** | .055 |
| Cubic | .011*** | .001 | .011*** | .001 | .010*** | .001 |  | .013*** | .001 | .013*** | .001 | .014*** | .001 |
| Δ_Linear_ _slope (a-non)_ | -.113 | .147 | -.099 | .147 | -.279 | .245 |  |  |  |  |  |  |  |
| Δ_Quadratic (a-non)_ | .050 | .061 | .045 | .061 | .144 | .082 |  |  |  |  |  |  |  |
| Δ_Cubic (a-non)_ | -.002 | .002 | -.002 | .002 | -.004* | .002 |  |  |  |  |  |  |  |
| **Latent variance**^a^ | | | | | | | | | | | | | |
| Intercept | .085*** | .016 | .074*** | .016 | .067*** | .018 |  | .185*** | .018 | .173*** | .017 | .151*** | .019 |
| Linear slope | .011*** | .002 | .011*** | .002 | .014*** | .003 |  | .017*** | .002 | .017*** | .002 | .019*** | .003 |
| **Intercept predicted by** | | | | | | | | | | | | | |
| Girl (0 = boy) |  |  |  |  | .130** | .049 |  |  |  |  |  | .201*** | .043 |
| Family SES |  |  |  |  | .001 | .001 |  |  |  |  |  | .000 | .001 |
| Immigrant (0 = no immigrant) |  |  |  |  | .086 | .062 |  |  |  |  |  | .094* | .045 |
| Prior achievement |  |  |  |  | -.048 | .046 |  |  |  |  |  | .055 | .047 |
| School-level achievement |  |  | .123*** | .033 | .154*** | .044 |  |  |  | .081** | .024 | .094** | .035 |
| **Linear slope predicted by** | | | | | | | | | | | | | |
| Girl (0 = boy) |  |  |  |  | -.074 | .076 |  |  |  |  |  | -.148* | .063 |
| Family SES |  |  |  |  | -.002 | .002 |  |  |  |  |  | -.001 | .002 |
| Immigrant (0 = no immigrant) |  |  |  |  | -.195* | .090 |  |  |  |  |  | -.190** | .067 |
| Prior performance |  |  |  |  | -.032 | .068 |  |  |  |  |  | -.018 | .067 |
| School- level achievement |  |  | -.131 | .135 | -.222 | .145 |  |  |  | .239* | .100 | .074 | .124 |
| Δ_LS_,_School-level ach. (a-non)_ |  |  | -.371* | .172 | -.295 | .193 |  |  |  |  |  |  |  |
| **Quadratic predicted by** | | | | | | | | | | | | | |
| Girl (0 = boy) |  |  |  |  | .022 | .019 |  |  |  |  |  | .030 | .016 |
| Family SES |  |  |  |  | .000 | .001 |  |  |  |  |  | .000 | .000 |
| Immigrant (0 = no immigrant) |  |  |  |  | .044* | .022 |  |  |  |  |  | .050** | .017 |
| Prior achievement |  |  |  |  | .012 | .018 |  |  |  |  |  | .006 | .017 |
| School-level achievement |  |  | .062 | .058 | .098 | .061 |  |  |  | -.096* | .042 | -.038 | .052 |
| Δ_Quadratic,School-level ach._ _(a-non)_ |  |  | .159* | .073 | .136 | .081 |  |  |  |  |  |  |  |
| **Cubic predicted by** |  |  |  |  |  |  |  |  |  |  |  |  |  |
| School-level achievement |  |  | -.002 | .002 | -.003 | .002 |  |  |  | .002 | .001 | .001 | .001 |
| Δ_Cubic,School-level ach._ _(a-non)_ |  |  | -.004* | .002 | -.004 | .002 |  |  |  |  |  |  |  |
| **Covariance** |  |  |  |  |  |  |  |  |  |  |  |  |  |
| Intercept with linear slope | .000* | .004 | .002 | .004 | -.003 | .004 |  | -.019** | .005 | -.018*** | .005 | -.018** | .006 |

*Note*. Prior performance = individual-level achievement in grade 6. School-level achievement = school-level achievement in grade 9. Nonacad. = nonacademic track. Δ_a-non_ = Difference between academic and nonacademic tracks for the specific parameter (i.e., mean, slope, or regression effect difference, respectively). Ach.= achievement

**p*<.05. ***p*<0.01. *** *p* <.001
